# Adhesion, Biofilm Formation, and Genomic Features of *Campylobacter jejuni* Bf, an Atypical Strain Able to Grow under Aerobic Conditions

**DOI:** 10.3389/fmicb.2016.01002

**Published:** 2016-06-30

**Authors:** Vicky Bronnec, Hana Turoňová, Agnès Bouju, Stéphane Cruveiller, Ramila Rodrigues, Katerina Demnerova, Odile Tresse, Nabila Haddad, Monique Zagorec

**Affiliations:** ^1^UMR 1014 SECALIM, OnirisNantes, France; ^2^Institute of Chemical Technology, Faculty of Food and Biochemical Technology, Department of Biochemistry and MicrobiologyPrague, Czech Republic; ^3^CNRS-UMR 8030 and Commissariat à l’Energie Atomique et aux Energies Alternatives CEA/DRF/IG/Genoscope LABGeMEvry, France

**Keywords:** food borne pathogen, biofilm, confocal microscopy, oxidative stress, genome sequence

## Abstract

*Campylobacter jejuni* is the leading cause of bacterial enteritis in Europe. Human campylobacteriosis cases are frequently associated to the consumption of contaminated poultry meat. To survive under environmental conditions encountered along the food chain, i.e., from poultry digestive tract its natural reservoir to the consumer’s plate, this pathogen has developed adaptation mechanisms. Among those, biofilm lifestyle has been suggested as a strategy to survive in the food environment and under atmospheric conditions. Recently, the clinical isolate *C. jejuni* Bf has been shown to survive and grow under aerobic conditions, a property that may help this strain to better survive along the food chain. The aim of this study was to evaluate the adhesion capacity of *C. jejuni* Bf and its ability to develop a biofilm. *C. jejuni* Bf can adhere to abiotic surfaces and to human epithelial cells, and can develop biofilm under both microaerobiosis and aerobiosis. These two conditions have no influence on this strain, unlike results obtained with the reference strain *C. jejuni* 81-176, which harbors only planktonic cells under aerobic conditions. Compared to 81-176, the biofilm of *C. jejuni* Bf is more homogenous and cell motility at the bottom of biofilm was not modified whatever the atmosphere used. *C. jejuni* Bf whole genome sequence did not reveal any gene unique to this strain, suggesting that its unusual property does not result from acquisition of new genetic material. Nevertheless some genetic particularities seem to be shared only between Bf and few others strains. Among the main features of *C. jejuni* Bf genome we noticed (i) a complete type VI secretion system important in pathogenicity and environmental adaptation; (ii) a mutation in the *oorD* gene involved in oxygen metabolism; and (iii) the presence of an uncommon insertion of a 72 amino acid coding sequence upstream from *dnaK*, which is involved in stress resistance. Therefore, the atypical behavior of this strain under aerobic atmosphere may result from the combination of insertions and mutations. In addition, the comparison of mRNA transcript levels of several genes targeted through genome analysis suggests the modification of regulatory processes in this strain.

## Introduction

*Campylobacter* is a Gram-negative bacterium, spiral-shaped and motile. This human pathogen lives as commensal of the gastrointestinal tract of most warm-blooded animals, especially poultry but also mammals (Park, 2002). Human infection by *Campylobacter* is commonly associated to the consumption of contaminated poultry meat. The genus *Campylobacter* includes very heterogeneous species that are present in a variety of environments but more than 80% of confirmed cases of campylobacteriosis were reported to be associated to *Campylobacter jejuni* (EFSA and ECDC, 2016).

The clinical manifestation of campylobacteriosis is severe gastro enteritis. However, *Campylobacter* infection is occasionally a precursor of serious post-infectious illness, including immune-reactive complications such as Guillain Barré and Miller Fisher Syndromes, two chronic and potentially fatal forms of paralysis (WHO, 2013). Since 2005, *Campylobacter* has been the most commonly reported human gastrointestinal bacterial pathogen in the European Union (EFSA and ECDC, 2016). In 2014, 236,851 cases of human campylobacteriosis were reported in EU. This zoonosis represents an incidence rate of 71 per 100,000 population exceeding the number of salmonellosis, which has a notification rate of 23.4 cases per 100,000 population. In addition, the cost of campylobacteriosis to public health systems and the loss of individual health and productivity were evaluated around 2.4 billion Euros per year in Europe (EFSA and ECDC, 2016) and between 1.2 and 4 billion $ for the US (Eberle and Kiess, 2012; Batz et al., 2014). The need for controlling this pathogen along the food chain explains the numerous studies reported in the literature that aimed at understanding its metabolism and virulence.

*Campylobacter jejuni* presents specific growth requirements, as it is thermotolerant with an optimal growth temperature of 40–42°C, microaerophilic (optimal O_2_ concentration of 5%), and capnophilic requiring 10% CO_2_ for an optimal growth. However, *C. jejuni* is able to persist in different environmental stress conditions explaining its high prevalence around the world. This food-borne pathogen has indeed developed adaptation mechanisms to survive under various harsh conditions it can encounter, from poultry gastrointestinal tract to the consumer’s plate. One of the most important characteristics of this bacterium is its ability to survive in aerobic environments despite its microaerophilic nature. This suggests an ability to cope with oxidative stress mediated by environmental oxygen tension and reactive oxygen species. To survive against such stresses, biofilm formation has been suggested to be one of the strategies used by this pathogen to persist in the environment (Buswell et al., 1998; Nguyen et al., 2012; Turonova et al., 2015). Commonly, biofilms are defined as multicellular layers of bacteria embedded within a matrix of extracellular polymeric substances (EPSs; Costerton, 1995; Costerton et al., 1995; Donlan, 2002; Donlan and Costerton, 2002). *C. jejuni* strains have been reported to be able to form different types of biofilm characterized as a structure attached to a surface, a pellicle formed at the surface of the liquid, or aggregates floating in the liquid culture (Joshua et al., 2006). Recently, we have reported the atypical property of *C. jejuni* Bf, a strain able to grow on plates under aerobic atmosphere, thus with a very low concentration of CO_2_ (0.035%), but with 21% O_2_ (Rodrigues et al., 2015). The possible growth of *C. jejuni* strains under aerobiosis and after various oxidative stresses was previously reported (Chynoweth et al., 1998; Garénaux et al., 2008b; Hinton, 2016). The aim of this study was to investigate the ability of *C. jejuni* Bf to adhere to biotic and abiotic surfaces and to form biofilm. We compared the behavior of this strain under both microaerobiosis and aerobiosis to determine a possible increased capacity to resist to the presence of high level of O_2_, which can be encountered during meat products processing and storage. Finally, genome comparison was also performed in order to detect genetic elements putatively involved in the phenotype of this strain. For that purpose, the draft genome (Bronnec et al., 2016) was completed and the gene and metabolic repertoires of *C. jejuni* Bf were compared to those of other complete or draft genomes.

## Materials and Methods

### Bacterial Strain and Culture Conditions

Stains used in this study are presented **Table 1**. *C. jejuni* strains were stored at -80°C in Brain Heart Infusion broth (BHI) containing 20% (vol/vol) glycerol. Prior to each experiment frozen cells were streaked on Karmali agar plates (Oxoid Limited, UK), incubated at 42°C for 24 h under microaerobic conditions in CampyGen sachet (Oxoid Limited, UK): 5% oxygen, 10% carbon dioxide, and 85% nitrogen.

**Table 1:** *Campylobacter jejuni* strains experimentally used this study.

Origin	Name^∗^	Source	Reference (published genome)
Clinical	*Cjj* NCTC 11168; ATCC 700819	Diarrheic patient	Parkhill et al., 2000; Gundogdu et al., 2007
	*Cjj* 81-176	Outbreak	Fouts et al., 2006, Unpublished
	*Cjd* 269.97	Bacteremia	Fouts et al., 2007, Unpublished
	*Cjj* 81116; NCTC 11828	Outbreak	Pearson et al., 2007
	*Cjj* 00-2538	Outbreak	Clark et al., 2014, Unpublished
	*Cjj* 00-2544	Outbreak	Clark et al., 2014, Unpublished
	*Cjj* 00-2426	Outbreak	Clark et al., 2014, Unpublished
	*Cjj* 00-2425	Outbreak	Clark et al., 2014, Unpublished
	*Cj* Bf	Campylobacteriosis	Bronnec et al., 2016
Meat	*Cj* RM1221	Skin of a retail chicken	Fouts et al., 2005
Poultry	*Cjj* 327	Turkey slaughterhouse	Takamiya et al., 2011
	*Cjj* 305	Turkey slaughterhouse	Takamiya et al., 2011
	*Cjj* DFVF1099	Chicken isolate	Takamiya et al., 2011
Cattle	*Cjj* ATCC 33560	Bovine feces	Zeng et al., 2013a

*Cjj: *Campylobacter jejuni* subsp. *jejuni*; *Cjd*: *Campylobacter jejuni* subsp. doylei; Cj: Campylobacter jejuni.

As described previously by Rodrigues et al. (2015), *C. jejuni* Bf cells can be acclimated to aerobic conditions (namely AAC cells for aerobically acclimated cells). This was performed by sub-culturing three times (once for 48 h and then twice 24 h) on Karmali agar plates under aerobiosis (air; Rodrigues et al., 2015). In order to maintain the same conditions for all samples, cultures under microaerobiosis were identically performed three times under microaerobiosis (MAC cells for microaerobic conditions).

### Adhesion to Inert Surfaces

The adhesion capability was evaluated using BioFilm Ring Test^®^ (BioFilm Control, France) as described by Sulaeman et al. (2010), with several modifications. Briefly, the experiments were performed using the kit commercialized by BioFilm Control (KITC004) including polystyrene Costar plates with flat bottom (Corning, USA), magnetic beads solution (TON004) and contrast liquid (LIC0001). Two conditions were tested for adhesion assay, microaerobiosis and aerobiosis. Grown cells were recovered from Karmali agar plates and suspended at 10^8^ CFU/mL (OD_610__nm_ = 0.5 ± 0.1) in filtered BHI (provided with the kit). *C. jejuni* suspensions (200 μL), containing magnetic beads at 1% (vol/vol), were inoculated in Costar plate wells. After 2 h of incubation at 42°C, the adhesion capability of strains was evaluated by measuring a biofilm formation index (BFI) with the BFC Element 3 software (BioFilm Control, France). Assays were repeated at least three times with three technical replicates.

### Confocal Laser Scanning Microscopy (CLSM)

#### Static Biofilm Formation Assay

*Campylobacter jejuni* Bf and *C. jejuni* 81-176 cells were recovered from Karmali agar plates and suspended in BHI at 10^8^ CFU/mL (OD_610__nm_ = 0.5 ± 0.1). Two hundred microliters of bacterial suspension were inoculated in sterile 96-well polystyrene microtiter plates with a micro-clear^®^ bottom 190 ± 5 μm (Greiner Bio One, Germany). Several incubation times (30 min, 1, 2, 4 h) at 42°C were tested to evaluate the minimum time required for adhesion of the cells at the bottom of the well. Adhesion was performed under microaerobiosis (with bacteria first grown under microaerobiosis) and under aerobiosis (with *C. jejuni* Bf grown under aerobiosis and *C. jejuni* 81-176 grown under microaerobiosis). Then, the bacterial suspension in the microtiter plate was carefully replaced with 200 μl of sterile BHI. Plates were then incubated at 42°C for 24 and 48 h under microaerobic or aerobic conditions. At least 1 h before the biofilm observation, the cells were stained by adding Syto 9 at 0.01 mM final concentration (LIVE/DEAD^®^ Kit, Life Technologies, USA) directly into the wells, following the method of Turonova et al. (2015). Experiments were performed using three biological replicates. For each condition, three technical replicates were performed, and two acquisitions in each of them.

#### Confocal Laser Scanning Microscopy

After staining, image acquisition was performed using a spinning disk confocal microscope (Andor, UK; Olympus, Japan). The entire wells were first inspected to see biofilm formation and its global structure. Two different locations of each well were scanned using a 10X objective lens with the signal recorded in the green channel (excitation 488 nm, emission 500–525 nm). The chosen place for the acquisition was representative of the whole structure and a stack of horizontal planar images with a size of x = 670.8 μm and y = 897.84 μm (e.g., 1040 × 1392 pixels) was scanned with a z-step of 1 μm. Video acquisitions were performed in a selected layer of the same size as described before using a 40X NA 1.4 oil immersion objective lens with an exposure time of 100 ms. Acquisitions were achieved in three distinct positions in the biofilm structure: the bottom, middle and top of the biofilm.

#### Image Processing

Confocal laser scanning microscopy (CLSM) images from top to bottom were processed using IMARIS software (v 7.6, Bitplane AG, Switzerland). For visualization of the biofilm, shadow projections and three-dimensional structures were generated. Beside the biofilm appearance, quantitative structural parameters of biofilms were calculated. Biofilm volume and thickness were the selected parameters used to compare the architectural differences of the biofilms formed. The bio-volume corresponds to the total volume of cells in the acquired field (x × y × z = μm^3^) and the thickness is the maximum height reached by the biofilm (μm).

### Adhesion Assay to Epithelial Intestinal Cells *In vitro*

Human intestinal cell lines HT29 and HT29-MTX were used to compare adhesion abilities of *C. jejuni* NCTC 11168, *C. jejuni* 81-176 and *C. jejuni* Bf under microaerobic conditions. In addition, adhesion capabilities of *C. jejuni* Bf acclimated to ambient air were also assessed. Maintenance of cells and adhesion assays were performed according to Haddad et al. (2010). Briefly, intestinal cells were grown in Dulbecco’s minimum essential medium (DMEM) supplemented with 10% fetal bovine serum (FBS), containing 200 mM L-glutamine, 250 μg/mL gentamicin (Sigma-Aldrich, USA) and 2.5 μg/mL amphotericin B (Sigma-Aldrich, USA). The cells were grown routinely in tissue culture flasks at 37°C in a 5% CO_2_-humidified atmosphere.

For experimental assays, cultured cells were dissociated from plastic flasks using trypsin-EDTA solution (Invitrogen, USA) and approximately 10^5^ eukaryotic cells were seeded into each well of 24-well tissues culture tray and incubated for 5 days at 37°C in humidified atmosphere at 5% of CO_2_. The cells were washed with DMEM and each well was inoculated with a suspension of approximately 10^7^ CFU of bacteria. To evaluate the number of adhered bacterial cells, the infected monolayers were incubated for 1 h at 37°C in a humidified 5% CO_2_ incubator and rinsed five times with phosphate buffered-saline (PBS, Eurobio, France). The cell monolayer was lysed by addition of 0.5 mL of Triton X-100 0.1% (Labo-Si, France) at room temperature for 30 min. *C. jejuni* cells were enumerated from the lysate on Karmali agar plates after 48 h incubation at 42°C under microaerobic condition. Experiments were performed using three biological replicates, and for each two technical replicates.

### Genome Sequence Completion and Comparative Genomic Analysis

To complete the draft genome sequence of *C. jejuni* Bf (Bronnec et al., 2016), PCR amplifications were performed on regions presenting uncertainties and for gap-filling purpose on contig extremities with primers designed in the flanking regions of each gap and PCR products were sequenced (Biofidal, France). As genome comparison showed that *C. jejuni* Bf was closer to other genomes than that of the reference genome of *C. jejuni* NCTC 11168 a new mapping was performed on the closest complete genome available (*C. jejuni* ATCC 32488 SRZ049709). Automatic annotation was performed on the MicroScope platform (MaGe; Vallenet et al., 2006, 2013) and manually checked.

Nucleotide sequence accession number: this whole genome project has been deposited in ENA under the accession no. FCEZ01000001-FCEZ01000095. The version described in this paper is the second version, FCEZ01000001-FCEZ01000095.

Using the tools available on the MicroScope platform, genomic comparisons were conducted between *C. jejuni* Bf genome and other *C. jejuni* genomes listed in Supplementary Table S1. A total of 33 complete and 19 draft *C. jejuni* genomes were used. “PkGDB Synteny Statistics” tool was used to perform similarity analysis between *C. jejuni* Bf and all *C. jejuni* genomes available to date on the PkGDB database. “Gene phyloprofile” tool has enabled the genomic comparison by searching specific genes of *C. jejuni* Bf in comparison with the other genomes, with the following homology constraints: minLrap ≥ 0.8, maxLrap ≥ 0 and identity ≥ 30%.

### RNA Isolation and Reverse Transcription

After growth AAC or MAC *C. jejuni* cells were recovered from Karmali plates and suspended in BHI at 10^8^ CFU/mL (OD_610__nm_ = 0.5 ± 0.1). RNA isolation, control and reverse transcription were performed according to Haddad et al. (2012) with some modifications. Briefly, one milliliter of this suspension was centrifuged at 3,300 *g* for 6 min at 4°C, and then resuspended in 1 mL of Extract-All (Eurobio, France) and mixed with 0.2 mL of chloroform. After a centrifugation at 12,000 *g* during 15 min at 4°C, RNAs from the aqueous phase were precipitated with isopropanol, washed twice in cold 75% ethanol and then solubilized in 50 μL of RNase-free water. Samples were then treated with TurboDNase (Life Technologies, France) to remove potential DNA contamination. The integrity of RNA was verified using 1% agarose gel and quantified using a NanoDrop spectrophotometer (Thermo Scientific, France). Absence of DNA contamination was validated by PCR. RNA was isolated from three biological replicates. Reverse transcription was performed on 100 ng of RNA using the RevertAid H Minus First-Strand cDNA synthesis kit (Euromedex, France) using random hexamer primers according to the manufacturer’s instructions.

### Quantitative Real-Time PCR

The quantitative real-time PCR assay was performed using SYBR Green I (Applied Biosystems, USA) and MJ Research PTC-200 Thermal Cycler (GMI, USA). The chosen internal control was *rrs* (Hyytiäinen et al., 2012) with primers rrs_F AAGGGCCATGATGACTTGACG and rrs_R AGCGCAACCCACGTATTTAG. The studied genes were *cosR* (with primers cosR_F TTTGAAAGCTGGAGCTGATG and cosR_R GGTTCCGCCAAGTCTTAGTC) and *dnaK* (DnaK)_F AAACGCCAAGCGGTAACTAA and DnaK_R TTCTTTAGCCGCGTCTTCAT). The operon *oorDABC* (with primers oorD2_F TGCGGTTTTAGGACAAATGA and oorD2_R TTCATCTCTTTTTGCCACCA, oorA2_F GCGGCAATGAGTGGAGTAAA and oorA2_R TTGGAAGACCTGTTGAAGGA, oorB2_F TGGTAAGTGGAGATGGGGATA and oorB2_R GTTGGGCTTGTTTGGGAAT, oorC_F GTGGTGGCCCTACTAAGGTG and oorC_R AACCCTTATCTGCAGTCGAAA) was also studied. Finally, a CDS of unknown function (u30002_F TTCAGAACCTACGAGGATGGA and u30002_R TTCAATCCTCCAAGCACACA) located upstream from *dnaK* was also investigated. The PCR mix was prepared as follows: 100 ng to 1 μg of cDNA (for *cosR* expression or *oorDABC, dnaK*, and *u30002_F*), 1 μM of each primers and 12.5 μL of SYBR Green I Master Mix. The amplification program included an initial denaturing step of 10 min at 95°C followed by 40 cycles of 15 s at 95°C and 1 min at 60°C. A negative control was included in each run. Relative quantification of gene expression was calculated according to the 2^-ΔΔCt^ method (Livak and Schmittgen, 2001). Results were normalized to the gene transcription of the reference strain *C. jejuni* 81-176 in microaerobic conditions. The experiments were performed in triplicate from three independent cultures. For each experiment, at least three technical replicates were realized.

### Statistical Analysis

Adhesion results from Biofilm Ring Test were analyzed using Statgraphics Centurion software version 17.1.06 (Statpoint Technologies, USA). An analysis of variance (ANOVA) was assessed to determine the individual effect of each variable (species and atmosphere). Statistical data were completed using the Fisher LSD (least significant difference) technique for multiple comparisons with a significance level at 95%.

Numerical data on biofilm formation obtained from IMARIS were also assessed for an ANOVA. The two variables identified were the maximum height of biofilm and the biomass volume. The two factors considered were the time of biofilm formation (24 or 48 h) and the combination strain/atmosphere, e.g., *C. jejuni* 81-176 grown under microaerobiosis (81-176_μO2_), *C. jejuni* Bf under microaerobiosis (Bf_μO2_) and *C. jejuni* Bf under aerobiosis (Bf_O2_). This procedure allows the analysis of variance at several factors for each variable. Significant effects were considered when *p*-value < 0.05.

Results obtained for the adhesion assay to epithelial intestinal cells *in vitro* and from RT-qPCR were analyzed using Student’s *t*-test. *p-*value < 0.05 were considered statistically significant.

## Results

### Adhesion Capability and Biofilm Ultrastructure to Abiotic Surfaces

#### Ability to Adhere to Abiotic Surface

**FIGURE 1:**
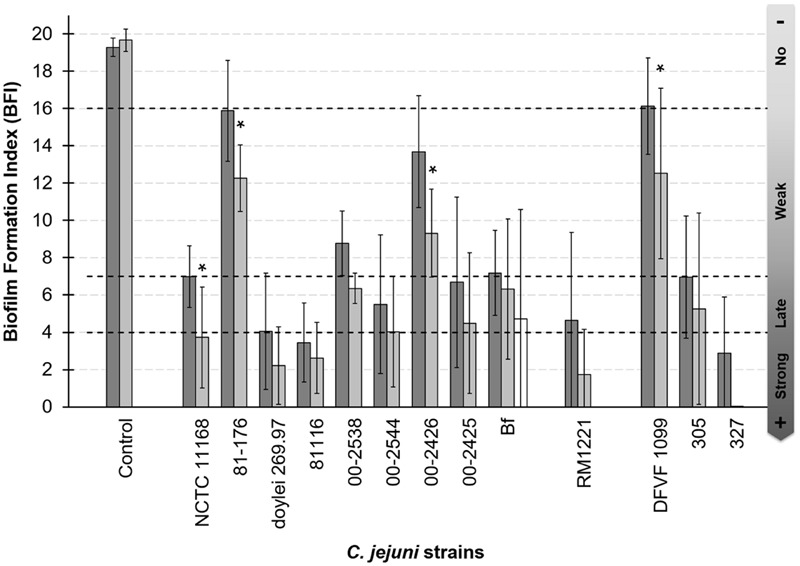
**Adhesion capability to polystyrene of *Campylobacter jejuni* is strain dependent.** Adhesion capability was measured after 2 h of incubation at 42°C under microaerobiosis with MAC grown cells (dark bars) and under aerobiosis with MAC grown cells (gray bars). White bar indicate biofilm formation index under aerobiosis of *C. jejuni* Bf AAC cells. The nine first strains were clinical isolates, the 10th was isolated from meat and three last strains were isolated from poultry. Four biological replicates were performed in microerobiosis and three in aerobiosis. Three technical replicate were realized each time. For the control and each strain, the mean values of BFI between O_2_ and μO_2_ conditions were compared. Asterisks show when these values were statistically different between the two conditions (*p*-value < 0.05).

Adhesion assays using BioFilm Ring Test^®^ method were conducted under microaerobic and aerobic conditions with an initial bacterial concentration of 5 × 10^6^ CFU/well. According to the biofilm formation index measured with the BFC Element 3 software all strains showed adhesion capacity and could be classified into four groups: strains with strong (0 ≤ BFI < 4), delayed (4 ≤ BFI < 7), or weak adhesion (7 ≤ BFI < 16), and those showing no adhesion capacity (BFI ≥ 16; **Figure 1**).

Among the 13 strains tested the ability to adhere to polystyrene varied independently from their clinical, animal, or food origin. Three strains were considered as strongly adherent (*C. jejuni* subsp. *jejuni* 81116, 327 and *C. jejuni* subsp. *doylei* 269.97), six showed a delayed adhesion (*C. jejuni* Bf, NCTC 11168, RM1221, 00-2544, 00-2425, and 305), and three presented a weak adhesion (*C. jejuni* 00-2538, 00-2426, 81-176). *C. jejuni* DFVF1099 appeared non-adherent under microaerobiosis. Although, the BFI values did not significantly differ between microaerobiosis and aerobiosis. Aerobiosis improved adhesion of *C. jejuni* NCTC 11168, 81-176, 00-2425 and DFVF (*p* < 0.05), and only a statistically non-significant tendency to better adhere was observed for the other strains. As among these strains, *C. jejuni* Bf is the only one able to grow on plate under aerobic condition (Rodrigues et al., 2015), the adhesion capability of cells grown under aerobiosis was also tested. As shown **Figure 1** *C. jejuni* Bf grown aerobically was able to adhere to inert surface as well as cells grown microaerobically, and the BFI did not statistically differed between these two conditions. Although, our adhesion results seemed contradictory with previous studies (Gunther and Chen, 2009; Sulaeman et al., 2010; Turonova et al., 2015), we chose to explore the capacity of biofilm formation of *C. jejuni* Bf in comparison to *C. jejuni* 81-176 because this virulent strain is consistently capable of producing mature biofilm (Gunther and Chen, 2009) and often considered as the reference. In addition, this strain could be used as a positive control for biofilm formation by CLSM and its well annotated genome was available.

#### Biofilm Development and Three-Dimensional Structure

We determined that a period of 2 h of adhesion to the polystyrene resulted in optimal initiation of biofilm formation for the two strains (data not shown).

**FIGURE 2:**
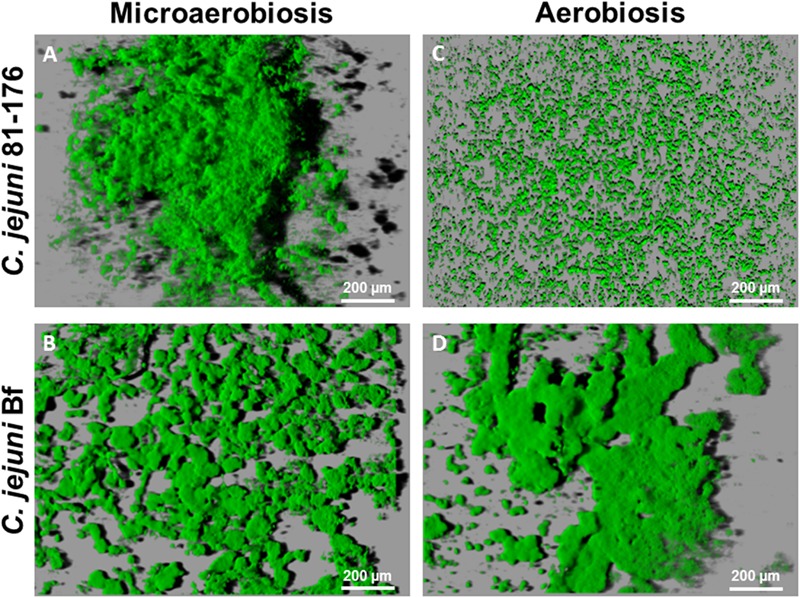
***Campylobacter jejuni* biofilm architecture after IMARIS processing of CLSM images from top to bottom.** Shadows projections were acquired for the biofilm developed after 24 h incubation at 42°C. Under microaerobiosis *C. jejuni* 81-176 **(A)** and *C. jejuni* Bf **(B)** established a biofilm. Under aerobiosis *C. jejuni* Bf **(D)** was able to develop a biofilm and *C. jejuni* 81-176 **(C)** persisted in its planktonic state.

After 24 h at 42°C under microaerobiosis, *C. jejuni* 81-176 developed a compact and highly structured biofilm strongly condensed at well center (**Figure 2A**, Supplementary Figure S1A). After 48 h of incubation the biofilm observed was quite similar with thick and dense structures (data not shown). Under the same conditions, *C. jejuni* Bf was also capable of forming biofilm but its structure seemed more expanded in the well and more flat in comparison with that of *C. jejuni* 81-176 (**Figure 2B**, Supplementary Figure S1B). The structure was less compact with a patchy coverage of the surface and composed by few large and compact structures and several microcolonies (**Figure 2B**, Supplementary Figure S1B).

During incubation under aerobiosis *C. jejuni* 81-176 did not develop any biofilm but rather, harbored microcolonies of surface attached cells (**Figure 2C**). In contrast *C. jejuni* Bf biofilm appeared more compact and structured under aerobic condition, as compared to the one formed in microaerobiosis (**Figure 2D**, Supplementary Figure S1C). After 48 h of cultivation at 42°C, biofilm formed by *C. jejuni* Bf was more compact with micro colonies less spread around the surface of the well (data not shown).

#### Quantification and Comparison of Biofilm Structures

**FIGURE 3:**
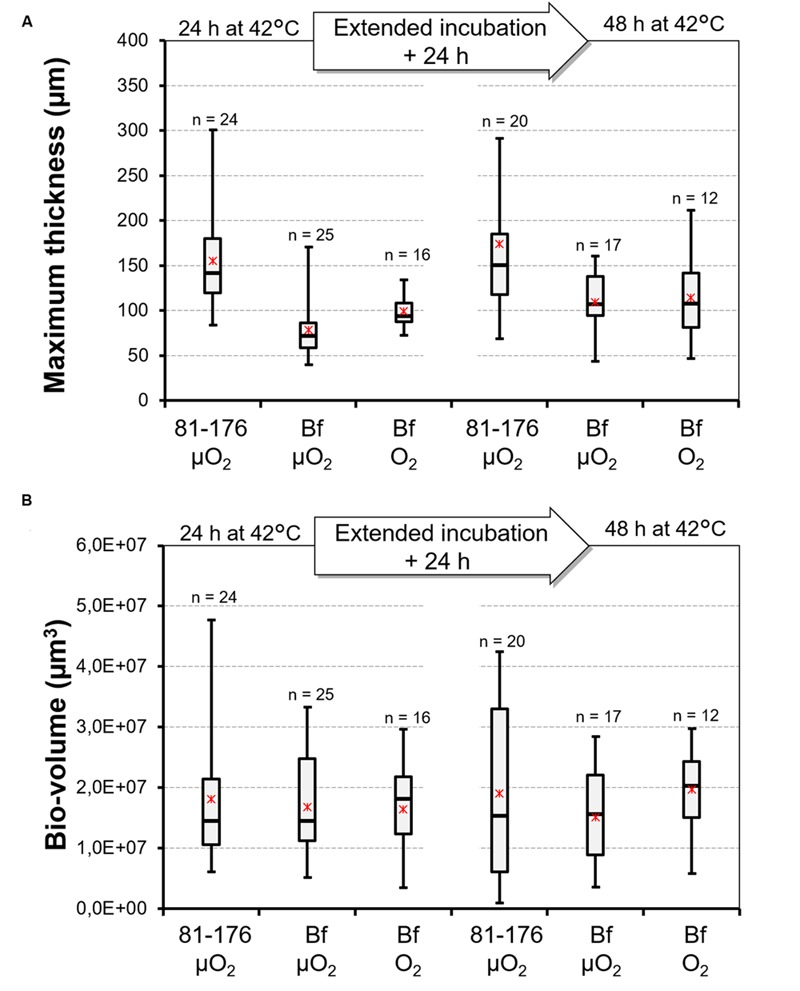
**Distribution of data describing the biofilm architecture of *C. jejuni* 81-176 and *C. jejuni* Bf.** Box plot representing the distribution of maximum thickness **(A)** and bio-volume **(B)** values observed for the biofilms developed by *C. jejuni* 81-176 and *C. jejuni* Bf under microaerobiosis (μO_2_) and under aerobiosis (O_2_) after 24 and 48 h of incubation at 42°C. Minimum and maximum values are reported. Asterisks indicate the mean and dashes the median. The number of repeats was added above each box plot.

The quantity of biofilm was characterized using two variables: bio-volume and maximum thickness. The individual effect of different factors (duration of cultivation, strain, atmosphere) on the two variables were considered (**Figure 3**). For each variable, the period of biofilm cultivation (24 or 48 h) had no significant effect. Multiple-comparison procedure was used to determine the significantly different means (Supplementary Table S2). For maximum thickness the Fisher’s LSD method revealed two significantly different groups T1 and T2. The first group (T1) encompasses biofilm structure formed by *C. jejuni* 81-176 and the second group (T2) is composed by biofilms formed by *C. jejuni* Bf under both microaerobic and aerobic conditions. Conversely, a unique homogeneous group (V) was obtained when considering biofilm volume, independently from the strain or the conditions tested.

#### Cell Motility Observation

As reported previously (Turonova et al., 2015), we observed motile *C. jejuni* 81-176 cells at different locations of the biofilm structure (e.g., at the bottom, middle, and top) after 24 and 48 h of biofilm formation. Similarly, a subpopulation of *C. jejuni* Bf also showed the capacity to move within the biofilm structure in the two conditions tested (Supplementary files S1 and S2). A better motility was detected at the bottom of the biofilm where the structure is more dispersed. No obvious difference was observed in the motility of *C. jejuni* Bf under microaerobiosis or aerobiosis.

### *C. jejuni* Bf Adhesion to Epithelial Intestinal Cells *In vitro*

**FIGURE 4:**
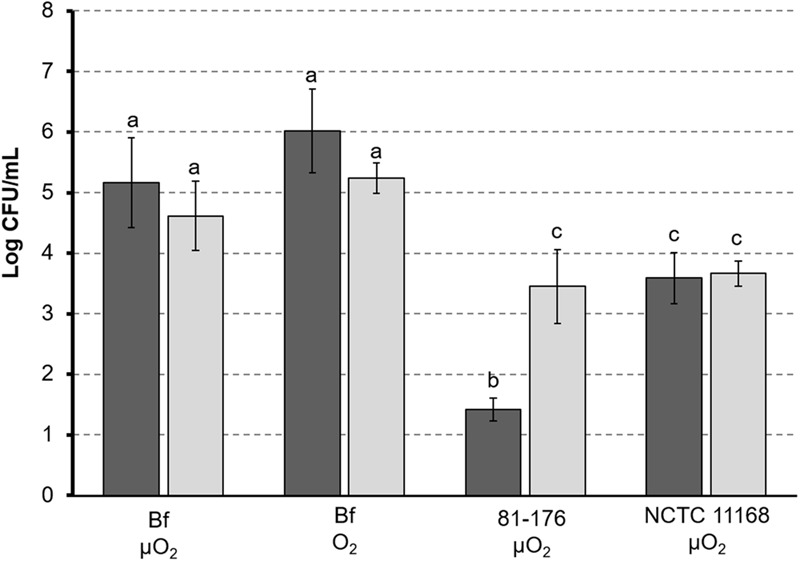
**Adhesion of *C. jejuni* to intestinal cells.** *Campylobacter jejuni* 81-176 and *C. jejuni* NCTC 11168 were grown under microaerobic conditions (μO_2_) and *C. jejuni* was cultured under microaerobic conditions (μO_2_) or ambient atmosphere (O_2_). Cells lines HT29 (black) and HT29-MTX producing mucus (gray) were used for adhesion tests. Adhesion of *C. jejuni* is expressed as amount of bacterial cells (expressed as CFU/mL) released from lysed eukaryotic cells after 1 h adhesion. Letters indicate values statistically similar (*p*-value < 0.05).

In addition to interaction with abiotic surfaces, we also determined the ability of *C. jejuni* Bf to adhere to biotic surfaces. For that purpose, the adhesion of *C. jejuni* Bf to HT29 and HT29-MTX cells was compared to those of *C. jejuni* 81-176 and NCTC 11168. The presence or absence of mucus did not significantly affect the adhesion of *C. jejuni* Bf and *C. jejuni* NCTC 11168 strains to intestinal cells (*p*-value < 0.05), whereas *C. jejuni* 81-176 adhered better to mucus producing cells (**Figure 4**).

Under microaerobic conditions, *C. jejuni* Bf exhibited a significantly (*p*-value < 0.05) higher adhesion capability than the two reference strains, independently on the cell line used for experiment (**Figure 4**). In addition, after growth under ambient atmosphere *C. jejuni* Bf showed the same adhesion properties than after growth under microaerobiosis (**Figure 4**).

### Genome Analysis

The analysis of the draft genome of *C. jejuni* Bf did not reveal any clear gene acquisition or deletion which could explain its ability to grow under aerobiosis (Bronnec et al., 2016). In the present study we completed the genome sequence and a deeper analysis of the gene repertoire of this strain was conducted. We first searched in the genome of *C. jejuni* Bf for functions that could potentially be involved in the singular phenotype of this strain: ability to grow, to adhere and to form biofilm independently from aeration conditions. A list of 165 *C. jejuni* genes reported in the literature as important for biofilm formation, adhesion, and oxygen metabolism was established (Supplementary Table S3) and their presence was searched in *C. jejuni* Bf genome. Some of these genes were putatively involved in several functions, also involved in adhesion to eukaryotic cells, or were reported to be affected by oxidative stress. Therefore, we considered them as significant for our study. Most of the literature dedicated to stress resistance and biofilm formation by *C. jejuni* focused on reference strains such as NCTC 11168, 81-176, and 81116. However, this species presents an important genomic diversity (Jeon et al., 2010; Zeng et al., 2013a). Therefore, we also compared the *C. jejuni* Bf genome sequence to 52 (complete or draft) *C. jejuni* genomes to search for genes that could be mutated or specific of *C. jejuni* Bf.

#### Gene Repertoire of *C. jejuni* Bf Related to Biofilm Formation and Adhesion

Many genes have been reported as directly or indirectly related to the biofilm development although the molecular mechanisms of their involvement are not clearly understood in *C. jejuni*. From various studies on *C. jejuni* we have selected 64 genes potentially required for strong biofilm formation and searched for their presence/absence in the genome of *C. jejuni* Bf. The results are presented Supplementary Table S3. Only four out of the 64 genes were missing in *C. jejuni* Bf. These correspond to CDS tagged as *cj0628, cj0755, cj1564*, and *cj1725* in *C. jejuni* NCTC 11168. The gene *cj0628* encodes CapA (*Campylobacter* adhesion protein A) an auto-transporter which was considered as an adhesin necessary for adhesion to Caco-2 cells and chicken colonization (Ashgar et al., 2007). The gene *cj0755* encodes the ferric enterobactin receptor CfrA and is overexpressed in *C. jejuni* NCTC 11168 biofilm cells but its absence has already been reported in other *C. jejuni* strains (Kalmokoff et al., 2006; Zeng et al., 2013a,b; Sung and Khan, 2015). Tlp3, a transducer-like protein recently renamed CcmL (Rahman et al., 2014) for *Campylobacter* chemoreceptor for multiple ligands is encoded by *cj1564*. A mutation of *ccmL* reduce motility and enhance biofilm formation in *C. jejuni* 11168-O (Rahman et al., 2014). These three genes and the putative periplasmic protein *cj1725*; also overexpressed in *C. jejuni* NCTC 11168 biofilm cells (Kalmokoff et al., 2006); are absent from *C. jejuni* Bf as previously reported for other *C. jejuni* genomes (Pearson et al., 2007; Hepworth et al., 2011).

A number of *Campylobacter* genes have been previously described as mediating *in vitro* adhesion to human cells. Most of these genes were present in *C. jejuni* Bf genome (Supplementary Table S3). Among those, genes encoding the fibronectin binding proteins CadF (Konkel et al., 1997; Ziprin et al., 1999; Monteville et al., 2003) and FlpA (Flanagan et al., 2009; Konkel et al., 2010), the adhesins PEB 1, PEB 4 (Kervella et al., 1993; Pei et al., 1998; Del Rocio Leon-Kempis et al., 2006; Asakura et al., 2007), and JlpA (Jin et al., 2001) were recorded in *C. jejuni* Bf. Moreover, the membrane proteins known to be involved in adhesion step, such as the major outer membrane protein MOMP, a porin (Moser et al., 1997), and KpsE involved in the export of the capsular polysaccharide (Bachtiar et al., 2007) were found on *C. jejuni* Bf genome. As well, the lipooligosaccharide (LOS) biosynthesis gene cluster composed of 14 genes flanked by *waaC*-*htrB* and *waaV*-*waaF* was also present. Moreover, the genes *cstII* and *neuBCA* responsible for the sialylation of LOS (Parker et al., 2005, 2008) were observed in the genome of *C. jejuni* Bf. Interestingly, *C. jejuni* Bf possesses the 13 genes encoding an entire type VI secretion system (T6SS; Bleumink-Pluym et al., 2013) firstly described in *C. jejuni* by Lertpiriyapong et al. (2012), including *hcp* and *icmF1* genes.

Although *C. jejuni* Bf possesses a large repertoire for adhesion and biofilm formation, some genes previously described as related to adhesion were absent from its genome. As mentioned above, the gene encoding the autotransporter protein CapA (Ashgar et al., 2007) is absent from *C. jejuni* Bf genome. In addition, the γ-glutamyltranspeptidase (GGT) involved in colonization of chicken is also absent from this strain. These genes are also absent in many *C. jejuni* isolates (Flanagan et al., 2009; Floch et al., 2014), for which the biofilm forming ability is yet unknown.

#### Gene Repertoire to Cope with Oxygen

Various enzymes and proteins are thought or known to protect bacteria against oxidative stress. Among them seven main enzymes/proteins and few regulators are well-documented in *C. jejuni* (Pesci et al., 1994; Grant and Park, 1995; Baillon et al., 1999; Ishikawa et al., 2003; Atack et al., 2008; Butcher et al., 2010; Hwang et al., 2011; Flint et al., 2014; Kim et al., 2015). These proteins involved in peroxide or superoxide detoxification include the alkyl hydroxyperoxide reductase (AhpC), the superoxide dismutase (SodB), the catalase (KatA) and Cj1386, the thiol peroxydase (Tpx), the bacterioferritin co-migratory protein (Bcp), and the bacterioferritin (Dps). The regulators Fur, PerR, and CosR have been reported to be involved in oxidative stress response. All the genes encoding enzymes or regulators involved in oxidative stress response are present in the genome of *C. jejuni* Bf (Supplementary Table S3).

A complete aerobic respiration pathway was detected with *ccoNOQP, petABC, cydAB nuoABCDEFGHIJKLMN*, and *sdhBC* gene clusters encoding cytochrome c oxidase, cytochrome bc and cytochrome bd complexes, NADH quinone oxidoreductase, and succinate dehydrogenase, respectively. As previously reported (Bronnec et al., 2016) the gene *oorD*, from the gene cluster *oorDABC* encoding 2-oxoglutarate oxidoreductase – a component of tricarboxylic acid (TCA) cycle – harbors a point mutation that may affect its activity. Since TCA cycle serves as electron donor for oxidative phosphorylation, we also search for genes involved in this metabolic route in *C. jejuni* Bf genome but did not notice any difference with other *C. jejuni* genomes (data not shown).

#### Comparative Genomics of *C. jejuni* Bf vs. Other Genomes

Comparing the gene repertoire of *C. jejuni* Bf with that of other strains, on the basis of the functions putatively involved in oxygen metabolism, biofilm formation and adhesion did not reveal any obvious missing gene in this strain. Therefore, we performed genome comparison without focusing on functions but rather to detect which strains were the closest, to narrow our analysis.

The genome similarity analysis was based on the number and percentage of identity of genes and on synteny groups. The comparison was realized using 52 genomes available (32 complete and 19 draft). We observed that *C. jejuni* Bf was divergent from the well-studied reference genomes (*C. jejuni* NCTC 11168 and *C. jejuni* 81-176). Among the other genomes included in our genomic comparison, *C. jejuni* ATCC 33560 draft genome was the closest. Interestingly, both strains belong to the same MLST group (Rodrigues et al., 2015; MLST database http://pubmlst.org/campylobacter). More than 98% of the CDS of *C. jejuni* Bf were in bidirectional best hits (BBHs) with the CDS of *C. jejuni* ATCC 33560 draft genome (34 contigs). Such a similarity between the two strains prompted us to compare their phenotype. *C. jejuni* ATCC 33560 was not aerotolerant (data not shown). Consequently, we focused on the differences between the genome sequences of these two strains. Thirty eight CDS were unique to the two strains compared to the 51 others strains, most of them considered as encoding peptides of unknown function (Supplementary Table S4). Among those we noticed a small CDS inserted in the cluster *hcrA*/*grpE*/*dnaK*, directly upstream of *dnaK*. This gene, of unknown function, encodes a protein of 72 amino acids that may potentially affect the expression of *dnaK*. Among the 37 remaining unique CDS, many were of small size and could be considered as false or doubtful CDS or resulting from fragmented genes. None could be associated to functions related to oxygen metabolism.

### Comparison of Gene Transcription in *C. jejuni* Bf under Different Atmospheres

The phenotype of *C. jejuni* Bf regarding growth, adhesion to biotic and abiotic surfaces and biofilm formation suggested that this strain behaves similarly under air or under atmosphere conditions described as optimal (low O_2_ concentration and high CO_2_ concentration). Since only few genome features specific to this strain were observed, we hypothesized that a subtle change in gene expression may be involved. According to the literature, CosR is involved in oxidative stress response but also in biofilm maturation in *C. jejuni* (Hwang et al., 2011, 2012, 2014; Oh and Jeon, 2014; Turonova et al., 2015). The expression of *cos*R from cells grown under microaerobic or aerobic condition was measured. As well we determined the expression of several genes that were pointed out during genome analysis: *oorDABC* genes, *dnaK* and its upstream CDS. *C. jejuni* 81-176 grown was used as a control. Under microaerobiosis, *cosR* and *oorDABC* gene expression levels in *C. jejuni* Bf were not statistically different from those of *C. jejuni* 81-176 whereas we noticed an 8-fold increase of *dnaK* expression in *C. jejuni* Bf.

After aerobic growth of *C. jejuni* Bf, the relative expression of *cosR* and *oorDABC* were strongly increased in comparison with *C. jejuni* Bf grown in microaerobiosis. Indeed, *cosR* expression level was 12-times higher in aerobiosis. As well, *oorD, oorA, oorB*, and *oorC* were expressed 22, 19, 18, and 12 times more, respectively. The expression of *dnaK* and its upstream CDS were constitutive in *C. jejuni* Bf whatever the conditions tested.

We searched for the presence of the CosR box previously reported in *C. jejuni* NCTC 11168 by Hwang et al. (2011, 2012) upstream from these genes. We observed a motif similar to the CosR box upstream from *oorD* with only 14 out of the 21 bp consensus sequence conserved. Interestingly, a similar box was also present upstream from *dnaK* due to the insertion of a small CDS. Although, the motif was moderately conserved (14 out of 21 bp) we cannot exclude that such an insertion in *C. jejuni* Bf may modify *dnaK* expression or regulation by comparison to *C. jejuni* 81-176.

## Discussion

During the last decade, *C. jejuni* has been regularly reported as the leading cause of bacterial foodborne infection in Europe. Given the public health significance of this zoonosis it is relevant to understand the survival mechanisms adopted by this pathogen. Indeed, passage through the food chain exposes this microaerophilic pathogen to various harsh environmental conditions including oxidative stress. Among the strategies to resist, biofilm is a life-style known to protect bacteria from various environmental stresses, antimicrobial agents and also increased bacterial resistance to host immune response (Gilbert et al., 1993; Donlan and Costerton, 2002; Chmielewski and Frank, 2003). Recently described, *C. jejuni* Bf presents a higher ability to survive against oxidative stress and this clinical strain also presents the particularity to grow under aerobic conditions (Rodrigues et al., 2015). In this report, we studied the ability of this strain to adhere and develop biofilms. We also evaluated the influence of aerobiosis on adhesion properties. Finally, we searched for genomic features that may explain the atypical phenotype of the strain.

Biofilm formation is a succession of several steps beginning with initial attachment. Therefore, we have investigated the capacity of *C. jejuni* to adhere to an inert surface in order to evaluate subsequently its ability to initiate and develop a biofilm. The adhesion capability was variable between the 13 strains we tested. *C. jejuni* Bf showed a delayed adhesion, suggesting that a longer contact period with the polystyrene may lead to a stronger adhesion. Surprisingly, *C. jejuni* 81-176 strain showed a low adhesion capacity, even after several verification tests, although, this strain was previously reported to adhere and develop biofilm (Gunther and Chen, 2009; Sulaeman et al., 2010; Turonova et al., 2015). The main differences between the current study and previous ones rely on the experimental design, especially the media used for growth. These have been already reported to influence *C. jejuni* adhesion to inert surface (Reeser et al., 2007).

We have also investigated the capacity of *C. jejuni* to adhere and form biofilm under aerobiosis. Interestingly, cultivation of *C. jejuni* Bf under aerobiosis enhanced its adhesion to polystyrene. Few studies have been conducted to evaluate the ability of *C. jejuni* to form biofilm aerobically (Asakura et al., 2007; Reuter et al., 2010; Turonova et al., 2015). As raised by Turonova et al. (2015, 2016) the use of CLSM allows observation of structural changes in the biofilm formed by *C. jejuni*. Subsequently to our adhesion assay, the capacity of *C. jejuni* Bf and *C. jejuni* 81-176 to produce biofilm under aerobiosis were also evaluated and observed using CLSM. The ultrastructure of the biofilm formed by *C. jejuni* 81-176 being well-characterized (Gunther and Chen, 2009; Turonova et al., 2015), we chose this stain as a reference. In optimal growth conditions (e.g., under microaerobiosis and at 42°C), *C. jejuni* Bf is also able to develop a structured biofilm as previously described for several *C. jejuni* strains (Asakura et al., 2007; Gunther and Chen, 2009; Reuter et al., 2010; Turonova et al., 2015). Comparison of bio-volume and thickness of the biofilm formed by the two strains cultivated in microaerobiosis revealed structural differences. Indeed, the biofilm developed by *C. jejuni* 81-176 appeared thick with heterogeneous structures, whereas the one formed by *C. jejuni* Bf was more homogeneous, flatter and spread in the well. Statistical analysis confirmed that *C. jejuni* 81-176 developed a biofilm 1.7 fold higher than *C. jejuni* Bf but with a non-significant difference in volume level. The microaerophilic strain *C. jejuni* 81-176 was unable to develop a biofilm in ambient atmosphere at 42°C even after 48 h of incubation. This apparent contradiction with other studies reporting that aerobiosis enhances biofilm formation may rely on differences in experimental conditions and on the strain that were used. Indeed most studies focused on *C. jejuni* NCTC 11168. These were performed under different growth conditions with the use of Brucella (Reuter et al., 2010) or Muller-Hinton broths (Asakura et al., 2007) and an incubation temperature of 37°C. The study including *C. jejuni* 81-176 was performed to compare only the influence of oxygen using O_2_ and CO_2_-enriched conditions, e.g., 19% O_2_, 10% CO_2_, 71% N_2_ (Turonova et al., 2015) which are different from the gaseous conditions we used (ambiant air). In addition, incubation temperature was 37°C and adhesion duration was longer (4–5 h; Turonova et al., 2015) vs. 42°C and 2 h in the present study.

*Campylobacter jejuni* Bf is able to develop biofilms under both microaerobiosis and aerobiosis, with no significant modification in terms of bio-volume and thickness. We can hypothesize that under aerobiosis *C. jejuni* Bf develops a more structured biofilm resulting in a microaerobic local environment more adequate for its growth, as was proposed for NCTC 11168 (Stewart and Franklin, 2008; Reuter et al., 2010; Turonova et al., 2015). Nevertheless, this study is the first report on the capacity of a *C. jejuni* strain to form biofilm after growth under aerobiosis.

Adhesion to surface is clearly a preliminary step to biofilm formation and some proteins involved in adhesion to inert surfaces are also important for interaction with epithelial cells. Compared to *C. jejuni* 81-176 and NCTC 11168, *C. jejuni* Bf presents a higher ability to adhere to human intestinal cells after growth in either microaerobiosis or aerobiosis. Mucus production did not modify adhesion capability of *C. jejuni* Bf and NCTC 11168, but enhanced that of *C. jejuni* 81-176. The better ability of the clinical strain *C. jejuni* Bf to adhere to human intestinal cells might be explained by the presence of a complete T6SS as reported in few other strains (Lertpiriyapong et al., 2012; Harrison et al., 2014; Corcionivoschi et al., 2015). This structure is absent from *C. jejuni* NCTC 11168 and 81-176.

Once the phenotype characterization performed, we focused on comparative genomics to point out genes specific of *C. jejuni* Bf. The genome analysis revealed that this strain possesses the genes necessary to develop a biofilm. Among all of the genes identified in the literature related to biofilm formation only four were absent, which is not particularly relevant since these genes are also absent from several *C. jejuni* genomes (Hofreuter et al., 2006; Rahman et al., 2014). In addition, we cannot totally exclude that their absence could result from sequencing errors or sequence misassembly. The gene repertoire of *C. jejuni* Bf necessary to resist to oxidative stress revealed no difference with that of other strains. In *C. jejuni* the CosR regulator has been reported as responsible for the regulation of genes participating to oxidative stress response but also to biofilm formation (Kalmokoff et al., 2006; Svensson et al., 2009; Garénaux et al., 2008a; Hwang et al., 2011, 2012; Oh and Jeon, 2014; Turonova et al., 2015). We have shown that *C. jejuni* Bf *cosR* was 12-fold over-expressed in aerobiosis, suggesting that the regulation of genes involved in oxidative stress response and biofilm formation might be modified in this strain. We highlighted two genetic modifications in *C. jejuni* Bf that may rely on its behavior: a point mutation in *oorD* (Bronnec et al., 2016) and an insertion upstream from *dnaK*. The *oorD* mutation may result in a different phenotype toward oxygen metabolism since in *Helicobacter pylori*, the 2-oxoglutarate oxidoreductase encoded by *oorDABC*, was reported as important for the microaerophilic phenotype of this species (Hughes et al., 1998). In addition, we showed that *C. jejuni* Bf *oorDABC* operon is up-regulated under aerobiosis. Conversely, *dnaK* transcription was constitutive in *C. jejuni* Bf regarding atmosphere used for growth. However, this gene is up-regulated in *C. jejuni* Bf, in comparison with 81-176. This may be the consequence of the insertion just upstream from *dnaK* which may result in a modification of its transcription. Furthermore, DnaK belongs to a protein family involved in general stress response. Its high level of expression in *C. jejuni* Bf might explain a better resistance to oxidative stress of this stain compared to that of *C. jejuni* 81-176. Comparing the resistance of the two strains to other stresses would be necessary to confirm this hypothesis. In addition, DnaK has also been described as moonlighting in several bacteria, i.e., harboring a different function when expressed on the cell surface (Amblee and Jeffery, 2015 and references therein). Indeed, DnaK from several Gram+ and Gram- species has been shown to bind plasminogen or eukaryotic cell surfaces when present on bacterial surface. We have no evidence of such a location in *C. jejuni* Bf, but this should be considered to search for a potential role of this protein, which gene is highly express in a clinical strain capable of adhering to surfaces and developing biofilm.

## Conclusion

The ability of *C. jejuni* to develop a structured biofilm is highly variable depending on the surface, the environmental conditions but also the strain. *C. jejuni* Bf has the particularity to multiply under aerobiosis, but we also have shown that this strain is able to form a structured biofilm when cultured in aerobic condition. Further experiments could be conducted at environmental temperatures (vs. optimal one, 42°C) to investigate *C. jejuni* Bf ability to form biofilm under aerobiosis. Genome analysis did not highlight any obvious acquisition of functions in this strain. Its atypical behavior apparently results from a modification in the regulation of several genes involved in oxidative stress response, oxygen metabolism, adhesion, and biofilm formation.

## Author Contributions

Conceived and designed the experiments: VB, NH, OT and MZ; performed the experiments: VB, HT, RR, AB and SC; analyzed the data: VB, MZ, NH, OT and SC; wrote the paper: VB, NH and MZ; corrected the paper: VB, NH, HT, OT, SC and MZ.

## Conflict of Interest Statement

The authors declare that the research was conducted in the absence of any commercial or financial relationships that could be construed as a potential conflict of interest.
