# Supplementary material for: Adhesion, Biofilm Formation, and Genomic Features of Campylobacter jejuni Bf, an Atypical Strain Able to Grow under Aerobic Conditions
Source: Front Microbiol. 2016 Jun 30;7:1002. doi: 10.3389/fmicb.2016.01002 (PMC4927563; doi:10.3389/fmicb.2016.01002)
Supplement: Supplementary file 1 [file Table_1.DOCX]

**Table S1. *C. jejuni* genome sequences used for genomic comparison**

| Sequence status | Name | Accession N° | |  |
| --- | --- | --- | --- | --- |
| Complete | *C. jejuni* subsp. *jejuni* IA3902 | | [NC_017279.1](http://www.ncbi.nlm.nih.gov/nuccore/NC_017279.1) | |
|  | *C. jejuni* subsp. *jejuni* 00-0949 | | NZ_CP010301.1 | |
|  | *C. jejuni* subsp. *jejuni* 00-1597 | | NZ_CP010306.1 | |
|  | *C. jejuni* subsp. *jejuni* 00-6200 | | NZ_CP010307.1 | |
|  | *C. jejuni* subsp. *jejuni* 01-1512 | | NZ_CP010072.1 | |
|  | *C. jejuni* 32488 | | NC_021834.1 | |
|  | *C. jejuni* subsp. *jejuni* 35925B2 | | NZ_CP010906.1 | |
|  | *C. jejuni* 4031 | | [NC_022529.1](http://www.ncbi.nlm.nih.gov/nuccore/NC_022529.1) | |
|  | *C. jejuni* subsp. *jejuni* CG8421 | | [NZ_CP005388.1](http://www.ncbi.nlm.nih.gov/nuccore/NZ_CP005388.1) | |
|  | *C. jejuni* subsp. *jejuni* F38011 | | [NZ_CP006851.1](http://www.ncbi.nlm.nih.gov/nuccore/NZ_CP006851.1) | |
|  | *C. jejuni* subsp. *jejuni* MTVDSCj20 | | [NZ_CP008787.1](http://www.ncbi.nlm.nih.gov/nuccore/NZ_CP008787.1) | |
|  | *C. jejuni* subsp. *jejuni* R14 | | [NZ_CP005081.1](http://www.ncbi.nlm.nih.gov/nuccore/NZ_CP005081.1) | |
|  | *C. jejuni* RM1221 | | NC_003912.7 | |
|  | *C. jejuni* RM1285 | | NZ_CP012696.1 | |
|  | *C. jejuni* subsp. *jejuni* RM3196 | | NZ_CP012690.1 | |
|  | *C. jejuni* subsp. *jejuni* RM3197 | | NZ_CP012689.1 | |
|  | *C. jejuni* subsp. *doylei* 269.97 | | NC_009707.1 | |
|  | *C. jejuni* subsp. *jejuni* 00-2425 | | NC_022362.2 | |
|  | *C. jejuni* subsp. *jejuni* 00-2426 | | NC_022352.2 | |
|  | *C. jejuni* subsp. *jejuni* 00-2538 | | NC_022351.2 | |
|  | *C. jejuni* subsp. *jejuni* 00-2544 | | NC_022353.2 | |
|  | *C. jejuni* subsp. *jejuni* 1336 | | [NZ_CM000854.1](http://www.ncbi.nlm.nih.gov/nuccore/NZ_CM000854.1) | |
|  | *C. jejuni* subsp. *jejuni* 414 | | [CM000855.1](http://www.ncbi.nlm.nih.gov/nuccore/CM000855.1) | |
|  | *C. jejuni* subsp. *jejuni* 81116 | | NC_009839.1 | |
|  | *C. jejuni* subsp. *jejuni* 81-176 | | NC_008787.1 | |
|  | *C. jejuni* subsp. *jejuni* ICDCCJ07001 | | [CP002029.1](http://www.ncbi.nlm.nih.gov/nuccore/CP002029.1) | |
|  | *C. jejuni* subsp. *jejuni* M1 | | [NC_017280.1](http://www.ncbi.nlm.nih.gov/nuccore/NC_017280.1) | |
|  | *C. jejuni* subsp. *jejuni* NCTC 11168 | | NC_002163.1 | |
|  | *C. jejuni* subsp. *jejuni* NCTC 11168-BN148 | | [NC_018521.1](http://www.ncbi.nlm.nih.gov/nuccore/NC_018521.1) | |
|  | *C. jejuni* subsp. *jejuni* PT14 | | [NC_018709.3](http://www.ncbi.nlm.nih.gov/nuccore/NC_018709.3) | |
|  | *C. jejuni* subsp. *jejuni* S3 | | [NC_017281.1](http://www.ncbi.nlm.nih.gov/nuccore/NC_017281.1) | |
|  | *C. jejuni* CJM1cam | | [NZ_CP012149.1](http://www.ncbi.nlm.nih.gov/nuccore/NZ_CP012149.1) | |
|  | *C. jejuni* subsp. *jejuni* YH001 | | [NZ_CP010058.1](http://www.ncbi.nlm.nih.gov/nuccore/NZ_CP010058.1) | |
| Draft | *C. jejuni* subsp. *jejuni* 2008-1025 | | AIOP01 | |
|  | *C. jejuni* subsp. *jejuni* 2008-894 | | AIOQ01 | |
|  | *C. jejuni* subsp. *jejuni* 2008-979 | | AIOU01 | |
|  | *C. jejuni* subsp. *jejuni* 260.94 | | AANK01 | |
|  | *C. jejuni* subsp. *jejuni* 305 | | ADHL01 | |
|  | *C. jejuni* subsp. *jejuni* 84-25 | | AANT02 | |
|  | *C. jejuni* subsp. *jejuni* BH-01-0142 | | ABKD01 | |
|  | *C. jejuni* subsp. *jejuni* CF93-6 | | AANJ01 | |
|  | *C. jejuni* subsp. *jejuni* CG8486 | | AASY01 | |
|  | *C. jejuni* subsp. *jejuni* DFVF1099 | | ADHK01 | |
|  | *C. jejuni* subsp. *jejuni* H22082 | | AEIP01 | |
|  | *C. jejuni* subsp. *jejuni* HB93-13 | | AANQ01 | |
|  | *C. jejuni* subsp. *jejuni* ICDCCJ07002 | | APNP01 | |
|  | *C. jejuni* subsp. *jejuni* ICDCCJ07004 | | APNQ01 | |
|  | *C. jejuni* subsp. *jejuni* LMG 9081 | | AIOM01 | |
|  | *C. jejuni* subsp. *jejuni* LMG 9872 | | AIPM01 | |
|  | *C. jejuni* subsp. *jejuni* NW | | AGTE01 | |
|  | *C. jejuni* X | | AVFM02 | |
|  | *C. jejuni* subsp. *jejuni* ATCC 33560 | | AIOL01 | |
