# Supplementary material for: Adhesion, Biofilm Formation, and Genomic Features of Campylobacter jejuni Bf, an Atypical Strain Able to Grow under Aerobic Conditions
Source: Front Microbiol. 2016 Jun 30;7:1002. doi: 10.3389/fmicb.2016.01002 (PMC4927563; doi:10.3389/fmicb.2016.01002)
Supplement: Supplementary file 2 [file Table_2.DOCX]

**Table S2. Statistical analysis of maximum thickness and bio-volume of biofilms formed by *C. jejuni* Bf and *C. jejuni* 81-176.**

Least squares means with 95% confidence intervals for the parameters maximum thickness of the biofilm and bio-volume are indicated for the biofilms form by the two strains grown under aerobiosis (O_2_) or microaerobiosis (µO_2_).

|  |  |  | **Maximum thickness (µm)** | | | | **Bio-volume (x 10^7^ µm^3^)** | | |
| --- | --- | --- | --- | --- | --- | --- | --- | --- | --- |
| **Incubation time** | **Strain of**  ***C. jejuni*** | **Atmosphere** | **Mean** | **SD** | **Groups**^a^ | **Groups**^a^ | **Mean** | **SD** | **Groups**^a^ |
| 24 hours | 81-176 | µO_2_ | 155.43 | 9.23 |  | T2 | 1.81 | 0.21 | V |
|  | Bf | µO_2_ | 78.29 | 9.04 | T1 |  | 1.68 | 0.21 | V |
|  | Bf | O_2_ | 99.01 | 11.30 | T1 |  | 1.68 | 0.26 | V |
|  |  |  |  |  |  |  |  |  |  |
| 48 hours | 81-176 | µO_2_ | 169.15 | 10.11 |  | T2 | 1.91 | 0.30 | V |
|  | Bf | µO_2_ | 106.40 | 10.96 | T1 |  | 1.51 | 0.25 | V |
|  | Bf | O_2_ | 99.61 | 13.05 | T1 |  | 1.97 | 0.30 | V |

^a^T1, T2, V form groups of means (Thickness or Volume) with no statistically significant differences (p value < 0.05).
