# Supplementary material for: Adhesion, Biofilm Formation, and Genomic Features of Campylobacter jejuni Bf, an Atypical Strain Able to Grow under Aerobic Conditions
Source: Front Microbiol. 2016 Jun 30;7:1002. doi: 10.3389/fmicb.2016.01002 (PMC4927563; doi:10.3389/fmicb.2016.01002)
Supplement: Supplementary file 4 [file Table_4.DOCX]

Table S4. Genes specific of *C. jejuni* Bf and *C. jejuni* ATCC 33560.

|  |  | **Id (Length)** | **Comment** | |
| --- | --- | --- | --- | --- |
| 1. **Genes present in *C. jejuni* Bf and *C. jejuni* ATCC 33560 and absent from other strains** | | | | |
|  | Operon 1 | *BOF_v2_150021* (355aa)^a^ | Putative N-6 DNA methyl transferase. 100% identity with *C. jejuni* ATCC 33650, *C. coli* H56 and *C. coli* LMG 23336, 64.3% with *C. concisus* UNSW2, 48-49% with different *Helicobacter*. | |
|  |  | *BOF_v2_150022* (237aa) | Putative restriction endonuclease. 100% identity with *C. jejuni* ATCC 33650, *C. coli* H56 and *C. coli* LMG 23336, 52.4% with *C. concisus* UNSW2, 42-44% *H. cinaedi* and *H. pylori.* | |
|  | Operon 2 | *BOF_v2_150032* (186aa)*BOF_v2_150033* (351aa) | *BOF_v2_150032*: Unknown function, no domain detected. 100% identity with *C. jejuni* ATCC 33650, 129-258, 1997-7, 41-44% with different *Helicobacter*.  *BOF_v2_150033*: Putative ATPase. 100% identity with *C. jejuni* ATCC 33650, 129-258, 1997-7, 67% with *C. lari* CF89-12, 31-38% with different *Helicobacter.* | |
|  | Operon 3 | *BOF_v2_80007* (83aa) *BOF_v2_80008* (38aa) *BOF_v2_80009* (85aa) *BOF_v2_80010* (345aa) | Unknown function, no domain detected. *BOF_v2_80007*: 98.8 % identity with *C. jejuni* ATCC 33560 (false CDS?). Contains DUF4365 but proteins in this family are typically between 182 and 530 amino acids in length.  *BOF_v2_80008*: orphan (false CDS?)  *BOF_v2_80009*: 98.8 % identity with *C. jejuni* ATCC 33560 (false CDS?) unknown function, no known domain  *BOF_v2_80010*: 100% identity with *C. jejuni* ATCC 33560 | |
|  | Operon 4 | *BOF_v2_870015* (314aa)*BOF_v2_870016* (706aa) | *BOF_v2_870015*: Unknown function. 100% identity with *C. jejuni* ATCC 33650, 99.04% with *C. jejuni* 1893 and 140-16, 86% with *C. coli*, identity score variable with different *Helicobacter*. Low GC% (26.88%). Contains DUF 1837. No visible domain association.  *BOF_v2_870016*: 100% identity with *C. jejuni* ATCC 33650, 98.39% with *C. jejuni* 1893 and 140-16, 52-55% with*C. coli* and few *Helicobacter*. Helicase superfamily. | |
|  | Operon 5 | *BOF_v2_u60012* (38aa)*BOF_v2_u60013* (298aa) | Unknown function, no domain detected.*BOF_v2_u60012*: 100% identity with *C. jejuni* ATCC 33560 and 97% with 3 *C. coli*, (false CDS?).*BOF_v2_u60013*: 100% identity with *C. jejuni* ATCC 33560 and 99% with 3 *C. coli*.. Fragmented gene (remnant?). | |
|  | Other genes | *BOF_v2_280004* (86aa) | Fragment of potassium-transporting ATPase B chain. Mutated *kdpB.* | |
|  |  | *BOF_v2_480035* (255aa) | Putative type II R-M system restriction endonuclease HinP1I. 100% identity with *C. jejuni* ATCC 33650, then <75% with some *Helicobacter* or other bacteria. In an operon with a methylase 100% identical to *C. jejuni* ATCC 33650, then <75% with some *Helicobacter* or other bacteria. Operon by itself unique to *C. jejuni* Bf and ATCC 33560 | |
|  |  | *BOF_v2_60006* (103aa) | *cdtBC* fragmented gene? Similar to Cytolethal distending toxin B from other C. jejuni. 100% identity on 103 aa with C. jejuni ATCC 33560 (123 aa) | |
|  |  | *BOF_v2_890005* (136aa) | Putative part of a transporter. 100% identity with *C. jejuni* ATCC 33650, 81% with *C. coli*. No domain detected but 50 aa of the C terminal part strongly homologous (80-85% identity) with part of an ABC transporter. CJBOF_v2_890006 being a membrane protein, and *CJBOF_v2_890004* being a putative transporter. | |
|  |  | *BOF_v2_90009* (95aa) | Unknown function, no domain detected. 99% identity with *C. jejuni* ATCC 33560, 66% with *C. coli.* | |
|  |  | *BOF_v2_930030* (47aa) | Unknown function, no domain detected. 100% identity with *C. jejuni* ATCC 33650, N terminal part (aa 1-31) identical with other *C. jejuni*. (47 aa false CDS?). CJBOF_v2_930029 being also a small peptide with homology to C terminal part of membrane protein of *C. jejuni*. Part of a truncated CDS? | |
|  |  | *BOF_v2_u680003* (54aa) | Unknown function, no domain detected. (Fragmented CDS?). Two insertions in a syntheny group with *C. jejuni* ATCC 33560. | |
|  |  | *BOF_v2_u150002* (38aa) | Unknown function, no domain detected (false CDS?) | |
|  |  | *BOF_v2_u170001* (96aa) | Unknown function, no domain detected. Identical to part of other *C. jejuni* genes (100% id to aa 129-224 *C. jejuni* ATCC 33560 and 4 other strains). u170003 43 aa long is unique to *C. jejuni* Bf, but false CDS? Fragmented CDS? | |
|  |  | *BOF_v2_u170006* (158aa) | Unknown function, no domain detected. N terminal aa 1-158, 95-100% identity with part of an uncharacterized protein from *C. jejuni* ATCC 33560, NW, and *C. coli* H8. | |
|  |  | *BOF_v2_u180002* (121aa) | Unknown function, no domain detected. N terminal aa 1-121, 100% identity with aa 1-121 of 224 aa long CDS of *C. jejuni* ATCC 33560, and 3 other *C. jejuni* strains and 1 *C. coli*. Fragmented CDS? | |
|  |  | *BOF_v2_u240002* (74aa) | Unknown function, no domain detected. 100% identity with *C. jejuni* ATCC 33560. 74 aa, false CDS? | |
|  |  | *BOF_v2_u370005* (42aa) | Unknown function, no domain detected. 42 aa 100% identity with *C. jejuni* ATCC 33560, and with part of other *C. jejuni* CDS. Truncated CDS? | |
|  |  | *BOF_v2_u600001* (125aa) | Unknown function. 125 aa 100% identity with *C. jejuni* ATCC 33560, 2 other *C. jejuni* and 2 *C. coli* strains. Belongs to DUF489 superfamily, with a PRK00218 domain (putative lysogenization regulator). No specific domain organization. Weak similarity with lysogenization proteins HflD of *Vibrios* | |
|  |  | *BOF_v2_u610005* (166aa) | Unknown function, no domain detected. 166 aa 100% identity with *C. jejuni* ATCC 33560 (9 aa in N terminus missing) and with few *C. coli*. aa 1-166 identical to part (aa 153-318) of a CDS annotated as methyltransferase samll from *C. jejuni* NCTC11351. | |
|  |  | *BOF_v2_u620006* (161aa) | Unknown function, no domain detected. 161 aa 100% identity with *C. jejuni* ATCC 33560, 82-99% some *C. jejuni* and *C. coli* strains. | |
|  |  | *BOF_v2_u620010* (283aa) | Unknown function. 283 aa long. aa 14-282 identical or similar to aa 1-269 (out of 349) with *C. jejuni* ATCC 33560 and 2 other *C. jejuni* and 1 *C. coli* strains. (2 N at the end of the sequence, at the end of a contig, may be part of a longer CDS). Contains ankyrin repeat domains (Eukaryotic ankyrin repeats mediate protein-protein interactions in very diverse families of proteins. The number of ANK repeats in a protein can range from 2 to over 20. Ankyrin repeats mediate protein-protein interactions in very diverse families of proteins. In bacteria (not in *C. jejuni* Bf) this ANK domain may be associated to uncharacterized protein domains conserved in bacteria (DUF2314). DUF2314 is found in various bacterial hypothetical proteins, as well as putative ankyrin repeat proteins. The exact function of the domains comprising this family has not, as yet, been determined. ANK domain can also be associated with a peptidase domain, or with domains involved in the regulation of 1,3-beta-glucan synthase activity and cell-wall formation. Genome contextual information showed that SMI1 are primary immunity proteins in bacterial toxin systems. ([Marchler-Bauer et al., 2015](#_ENREF_1)) | |
|  |  | *BOF_v2_820001* (127aa) | Unknown function, no domain detected. aa 1-127 identical to aa 1-127 (out of 305) of a CDS annotated as alpha-2,3-sialyltransferase from *C. jejuni* ATCC 33560. Fragmented CDS? | |
|  |  | *BOF_v2_u180007* (663aa) | Unknown function, no domain detected. Similar to an uncharacterized protein fo *C. jejuni* LMG 23263, 87459, and 51037. | |
|  |  | *BOF_v2_u180009* (60aa) | Unknown function, no domain detected. 98-100% identity with an uncharacterized protein from *C. jejuni* ATCC 33560, LMG 23269, LMG 23210, 1997-10, 2008-979, and *C. coli* 2548. Also conserved in *H. pylori*. | |
|  |  | *BOF_v2_u30002* (72aa) | Unknown function. 98.6% identity with an uncharacterized protein from C. jejuni ATCC 33560 and similar (33-36% identity) to uncharacterized protein from Brachyspira murdochii. Possesses (aa 39-69, out of 72 aa) a SGNH_hydrolase, or GDSL_hydrolase domain (diverse family of lipases and esterases). In eukaryotes,PAF_acetylhydrolase (PAF-AH)_like subfamily of SGNH-hydrolases are key players in inflammation. PAF-AH is a calcium independent phospholipase A2 which exhibits strong substrate specificity towards PAF, hydrolyzing an acetyl ester at the sn-2 position. PAF-AH also degrades a family of oxidized PAF-like phospholipids with short sn-2 residues. In addition, PAF and PAF-AH are associated with neural migration and mammalian reproduction ([Marchler-Bauer et al., 2015](#_ENREF_1))**.** | |
|  |  | BOF_v2_u610004 (216aa) | Unknown function. 100% identity with an uncharacterized protein from *C. jejuni* ATCC 33560 and *C. coli* 132-6. Possesses DUF1911 domain found in a set of hypothetical bacterial proteins. | |
|  |  | *BOF_v2_u690010* (58aa) | Unknown function, no domain detected. 100% identity with part of an uncharacterized protein from several *C. jejuni* strains. Truncated CDS? | |
|  |  | BOF_v2_10001 (73aa) | Unknown function, no domain detected. 100% identity with an uncharacterized protein from several *C. jejuni* strains. | |
|  |  | *BOF_v2_890007* (278aa) | Unknown function. 100% identity with an uncharacterized protein from *C. jejuni* ATCC 33560, and 76.7% with *C. coli* LMG 9860 and 151-9, < 30% identity in other *C. jejuni* strains.Possesses a DUF342 domain (aa 162-259) and with aa 171-208 a multidomain of Tektin family. Tektins are cytoskeletal proteins demonstrated ciliary and flagellar doublet microtubules. Tektins form unique protofilaments, organized as longitudinal polymers of tektin heterodimers with axial periodicity matching tubulin. Tektin polypeptides consist of several alpha-helical regions that are predicted to form coiled coils. Tektins share structural similarities with intermediate filament proteins. Possible functional roles for tektins are stabilization of tubulin. ([Marchler-Bauer et al., 2015](#_ENREF_1)). | |
| 1. **Genes specific of *C. jejuni* ATCC 33560 and absent from *C. jejuni* Bf** | | | | |
|  |  | *AIOL_v1_1150004* (47aa) | | No putative conserved domains have been detected. Present in other *C. jejuni* strains. Truncated ? |
|  |  | *AIOL_v1_160002* (119aa) | | Cytoplasmic. Present in other *C. jejuni* strains. No putative conserved domains have been detected |
|  |  | *AIOL_v1_2170001* (30aa) | | Unknown function, no domain detected. Orphan? false CDS? |
|  |  | *AIOL_v1_30067* (40aa) | | Unknown function, no domain detected. Orphan? false CDS? |
|  |  | *AIOL_v1_40013* (56aa) | | Unknown function, no domain detected. Orphan? false CDS? |
|  |  | *AIOL_v1_40062* (183aa) | | Similar to Lipopolysaccharide core biosynthesis putative protein. 99.45% identity, on 183 aa; from *C. jejuni* 81-176), and 99.4 %, on 167 aa with *C. jejuni* 129-258 |
|  |  | *AIOL_v1_630004* (39aa) | | Unknown function, no domain detected. 39 aa. 100% identity with an uncharacterized protein from *C. jejuni* 1336, 51037, and 51494. |
|  |  | *AIOL_v1_630007* (49aa) | | Unknown function, no domain detected. 49 aa. 92.1% identity with an uncharacterized protein from *C. coli.* |
|  |  | *AIOL_v1_660020* (40aa) | | Partial ABC transporter? 100% identity on part (40 aa) of an ATP-binding protein of ABC transporter from *C. jejuni* 1997-11 and HB93-13. |
|  |  | AIOL_v1_750010 (21aa) | | Unknown function, no domain detected. Orphan? false CDS? |

^a^Length in amino acid (aa) is indicated

Marchler-Bauer, A., Derbyshire, M.K., Gonzales, N.R., Lu, S., Chitsaz, F., Geer, L.Y., et al. (2015). CDD: NCBI's conserved domain database. Nucleic Acids Res. 43**,** D222-226. doi: 10.1093/nar/gku1221.
