# Supplementary material for: Adhesion, Biofilm Formation, and Genomic Features of Campylobacter jejuni Bf, an Atypical Strain Able to Grow under Aerobic Conditions
Source: Front Microbiol. 2016 Jun 30;7:1002. doi: 10.3389/fmicb.2016.01002 (PMC4927563; doi:10.3389/fmicb.2016.01002)
Supplement: Supplementary file 5 [file Presentation_1.PPTX]

## Slide 1
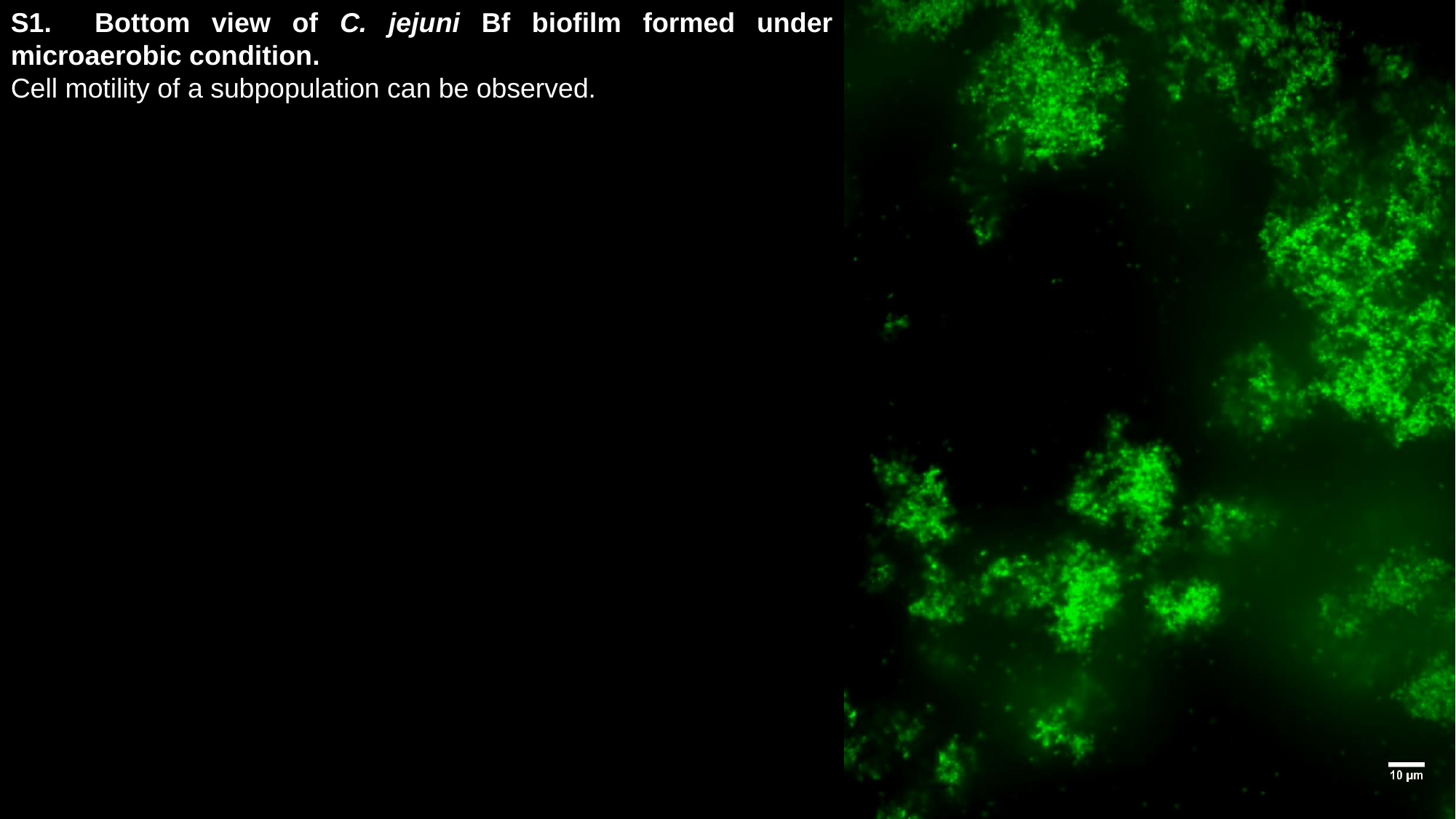

S1. Bottom view of C. jejuni Bf biofilm formed under microaerobic condition.
Cell motility of a subpopulation can be observed.
