# Supplementary figures and images for: Adhesion, Biofilm Formation, and Genomic Features of Campylobacter jejuni Bf, an Atypical Strain Able to Grow under Aerobic Conditions
Source: Front Microbiol. 2016 Jun 30;7:1002. doi: 10.3389/fmicb.2016.01002 (PMC4927563; doi:10.3389/fmicb.2016.01002)

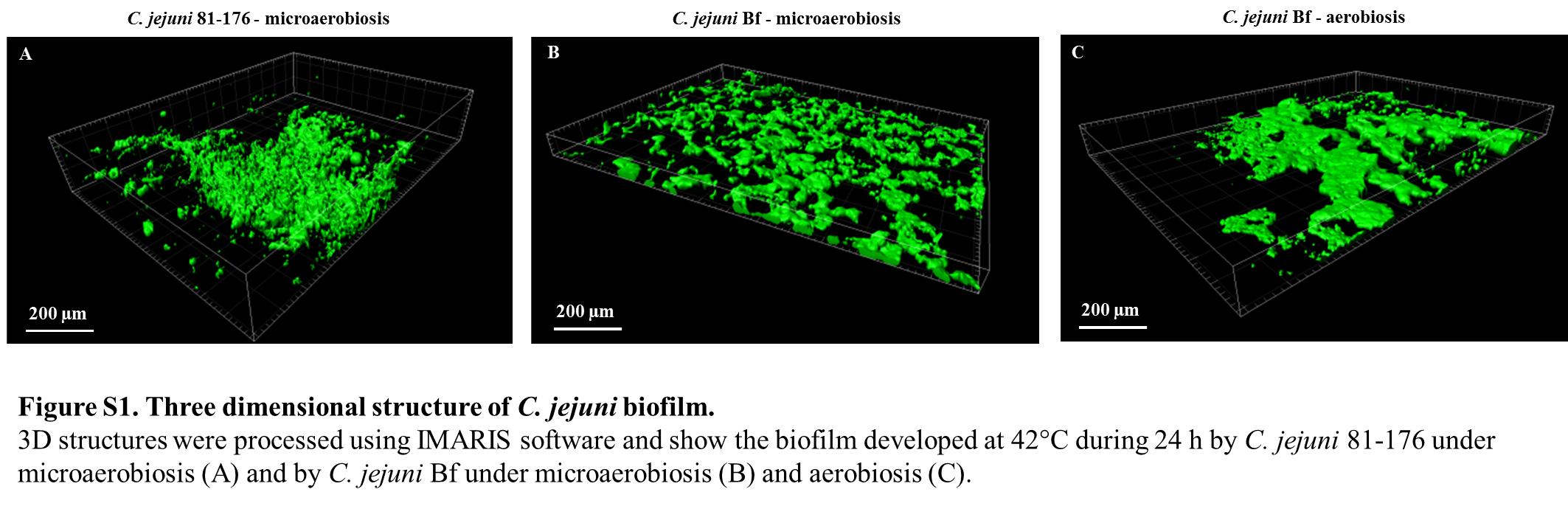

Supplement: Supplementary file 7 [file Image_1.TIF]
